# Altered neurovascular coupling in patients with vascular cognitive impairment: a combined ASL-fMRI analysis

**DOI:** 10.3389/fnagi.2023.1224525

**Published:** 2023-06-21

**Authors:** Zhao Ruan, Dong Sun, Xiaoli Zhou, Minhua Yu, Sirui Li, Wenbo Sun, Yidan Li, Lei Gao, Haibo Xu

**Affiliations:** ^1^Department of Radiology, Zhongnan Hospital of Wuhan University, Wuhan, Hubei, China; ^2^Department of Neurology, Zhongnan Hospital of Wuhan University, Wuhan, Hubei, China

**Keywords:** vascular cognitive impairment, cerebral small vessel disease, post-stroke cognitive impairment, neurovascular coupling, white matter lesion

## Abstract

**Background and objective:**

This study aims to examine the role of neurovascular coupling (NVC) in vascular cognitive impairment (VCI) by investigating the relationship between white matter lesion (WML) burden, NVC, and cognitive deficits. Additionally, we aim to explore the potential of NVC as a tool for understanding the neural mechanisms underlying VCI.

**Methods:**

This study included thirty-eight small vessel disease cognitive impairment (SVCI) patients, 34 post-stroke cognitive impairment (PSCI) patients, and 43 healthy controls (HC). Comprehensive assessments, including neuroimaging and neuropsychological testing, were conducted to evaluate cognitive function. WML burden was measured and correlated with NVC coefficients to examine the relationship between white matter pathology and NVC. Mediation analysis was employed to explore the link relationship between NVC, WML burden, and cognitive function.

**Results:**

The present study showed that NVC was significantly reduced in the SVCI and PSCI groups compared with HCs at both whole-brain and brain region level. The analysis revealed notable findings regarding NVC in relation to WML burden and cognitive function in VCI patients. Specifically, reduced NVC coefficients were observed within higher order brain systems responsible for cognitive control and emotion regulation. Mediation analysis demonstrated that NVC played a mediating role in the relationship between WML burden and cognitive impairment.

**Conclusion:**

This study reveals the mediating role of NVC in the relationship between WML burden and cognitive function in VCI patients. The results demonstrate the potential of the NVC as an accurate measure of cognitive impairment and its ability to identify specific neural circuits affected by WML burden.

## Introduction

With increased human longevity, dementia has emerged as a prominent health issue among the elderly. Cerebrovascular disease represents the second leading cause of dementia (Toledo et al., 2013). The prevalence of vascular cognitive impairment (VCI) ranges 15–40% in individuals over the age of 65 (van der Flier et al., 2018). VCI encompasses a wide range of cognitive impairment, from mild vascular cognitive impairment to vascular dementia (VaD) (van der Flier et al., 2018). The most common cause of VCI is cerebral small vessel disease (CSVD) (Iadecola et al., 2019), while stroke also contributes significantly to VCI (Lo et al., 2019). Both CSVD and stroke impact cerebral blood flow (CBF) regulation, metabolic demand, and neuronal activity (Wallin et al., 2018). However, the neural mechanisms underlying VCI remain incompletely understood.

Neurovascular coupling (NVC) is the temporal coordination between CBF and neural activity. This concept has a long history, originating from the work of Dutch psychologist Donders (Phillips et al., 2016). NVC is essential for maintaining normal brain function, with the neurovascular unit playing a key role in regulating blood supply and cellular processes to maintain homeostasis between neurons, glial cells, and vascular cells (Attwell et al., 2010). In cerebrovascular diseases, including CSVD, disruption of the neurovascular unit is often referred to as “neurovascular decoupling” (Iadecola, 2013; Shabir et al., 2018). Numerous studies have shown a strong correlation between neurovascular decoupling and cognitive impairment (Tarantini et al., 2019), although most of these studies have been conducted in animal experiments (Tucsek et al., 2014; Toth et al., 2017).

Non-invasive imaging techniques are urgently needed to investigate NVC. Liang et al. (2013) established a link between CBF and intrinsic brain activity measured by resting-state functional magnetic resonance imaging (rs-fMRI), suggesting that this correlation reflects NVC. Studies integrating neurophysiology and rs-fMRI have demonstrated a close relationship between the amplitude of low-frequency fluctuations (ALFF) in blood oxygen level-dependent (BOLD) signals (Zang et al., 2007) and spontaneous neuronal activity (Logothetis et al., 2001; Nir et al., 2007). Arterial spin labeling (ASL), a technique utilizing endogenous water molecules as diffusion markers, provides valuable insights into CBF and disease pathogenesis. Reduced CBF, associated with the extent of cognitive impairment, has been observed in VCI (Sun et al., 2016). The integration of ASL-derived cerebral perfusion information and rs-fMRI-derived cerebral neural function, along with their coupling analysis, has gradually gained traction in the field of cognitive impairment research. This approach has been utilized in several neurological disorders such as end-stage renal disease (Li et al., 2021), chronic migraine (Hu et al., 2019), and schizophrenia (Zhu et al., 2017), as well as cerebrovascular diseases such as hypertension and ischemic stroke, demonstrating associations with NVC dysfunction and cognitive impairment (Rousseaux et al., 2010; Bloch et al., 2015). These findings suggest that NVC may serve as a physiological basis for cognitive function and can be studied in VCI patients using this approach.

Neurovascular coupling has been chosen as the focus of this study due to its unique advantages in VCI researches. It serves as a bridge between vascular pathology and cognitive decline by capturing the dynamic relationship between neuronal activity and CBF (Sanford et al., 2022). Unlike other measures, NVC detects early changes in cerebral perfusion and neural activity, providing a non-invasive assessment of neurovascular regulation. Impaired NVC in VCI leads to a mismatch between metabolic demand and blood supply, resulting in cognitive impairment (Yabluchanskiy et al., 2020). NVC also integrates functional and physiological aspects of brain function, simultaneously assesses CBF and spontaneous neural activity. This holistic approach goes beyond cognitive assessments alone, which may miss physiological changes (Hu et al., 2019). Moreover, NVC is highly sensitive to subtle alterations in cerebral perfusion and neuronal activity (Claassen et al., 2021), enabling early diagnosis and monitoring in the heterogeneous and subtle changes seen in VCI.

Several studies have investigated NVC in VCI using various neuroimaging techniques. For instance, Liu et al. (2021) demonstrated disrupted NVC in patients with subcortical ischemic vascular disease, showing a mismatch between regional cerebral blood flow and neuronal activity. Similarly, Shabir et al. (2018) reported impaired NVC in patients with small vessel disease, highlighting the association between reduced NVC and cognitive decline. These studies collectively emphasize the significance of NVC dysfunction in VCI pathogenesis. Accumulating evidence highlights the role of the NVC in influencing an individual’s cognitive abilities. However, it is important to note that previous research in this field has primarily focused on studying specific subtypes of VCI, thereby overlooking the diverse subtypes that VCI encompasses. Furthermore, an important factor that has been relatively neglected in these studies is the presence of white matter lesions (WML), which has intricate connections with both NVC and VCI. WML represent a prevalent manifestation of cerebral small vessel lesions observed in neuroimaging, capable of eliciting cognitive impairment and other associated symptoms (Sarbu et al., 2016). However, recent studies have demonstrated incongruent effects of WML volume and location on cognitive impairment in patients (Kim et al., 2015; Veselý and Rektor, 2016). Moreover, WML shows a strong association with NVC (Sorond et al., 2013). Therefore, the interactions between WML, NVC, and VCI are worth studying.

To investigate whether NVC dysfunction is a potential mechanism leading to VCI, to identify new imaging markers of VCI and to investigate the interaction of WML, NVC, and VCI, this study performed a broad series of multimodal neuroimaging analyses to test three hypotheses. First, we hypothesized that patients with VCI would have impaired NVC. Second, we hypothesized that NVC dysfunction would be associated with cognitive decline. Finally, we hypothesized that NVC dysfunction would act as a mediator in the relationship between clinical features of VCI and cognitive impairment. To test these hypotheses, the main objectives of this study were to determine changes in regional ALFF and CBF in patients with VCI, to assess the extent of NVC changes, and to examine the mediating role of NVC in the relationship between WML burden and cognitive performance. NVC coefficients calculated from regional ALFF and CBF measures were used in a mediation model to examine the role of NVC in the relationship between WML burden and global cognition in patients with VCI.

## Subjects and methods

### Participants

This study received approval from the Ethical Medicine Society of Zhongnan Hospital of Wuhan University, and all participants provided informed consent by signing a consent form. Given that small vessel cognitive impairment (SVCI) and post-stroke cognitive impairment (PSCI) constitute the majority of VCI (Skrobot et al., 2018; Iadecola et al., 2019), the inclusion of SVCI and PSCI patients ensures adequate representation. A total of 72 VCI patients (38 SVCI and 34 PSCI) were recruited from outpatient and inpatient settings between December 2019 and October 2021. Additionally, 43 demographically matched healthy controls (HCs) were enrolled during the same period. Detailed inclusion and exclusion criteria are as follows.

### Inclusion and exclusion criteria

Inclusion criteria: (1) SVCI: at least one acute ischemic cerebrovascular disease has been onset for more than 3 months; PSCI: the onset time of stroke patients is within 6 months; confirm that the stroke through medical history and medical imaging and rule out hemorrhagic stroke; and neuroimaging findings of these two subtypes meeting the VICCS-2 neuroimaging criteria (Skrobot et al., 2018); (2) age 40–70 years; (3) meet the diagnostic criteria for VCI (Skrobot et al., 2018); (4) able to cooperate in completing various instrumental examinations and neuropsychological scale tests; and (5) voluntary signing of informed consent.

Exclusion criteria: (1) with severe heart, brain, lung, and kidney diseases; (2) with vision and hearing impairment that significantly affect cognitive testing; (3) With a history of psychoactive substance abuse; (4) other severe physical diseases that affect cognitive testing, such as coma, epilepsy, hypothyroidism, hypoxemia, etc.; (5) with a history of mental illness; and (6) with contraindication to MRI, and is unwilling to participate research and unconditional follow-up.

### Neuropsychological assessment

All participants underwent the following neuropsychological assessments: (1) neuropsychiatric inventory (NPI), and (2) global cognition: the mini-mental state examination (MMSE) (Tombaugh and McIntyre, 1992) and the Montreal Cognitive Assessment (MoCA) (Nasreddine et al., 2005).

### MRI data acquisition

One hour following the completion of the neuropsychological assessment, imaging data were acquired using a 3.0 T MR scanner (Discovery 750 w, GE Healthcare, Waukesha, WI, USA) equipped with a 32-channel head coil. The imaging protocol encompassed high-resolution T1 and T2-FLAIR scans (176 sagittal slices, 1-mm in-plane resolution), rs-fMRI (TR/TE = 2000/30 ms, voxel size 3.75 mm × 3.75 mm × 3.75 mm, 40 slices) with a duration of 8 min and 10 s, consisting of 185 functional volumes, and a background-suppressed three-dimensional pseudo-continuous arterial spin labeling (PCASL) sequence (TR/TE = 10.5/4.9 ms, flip angle 111°, 50 slices, slice thickness 4 mm without gap, field of view (FOV) 240 mm × 240 mm, matrix size 128 × 128, labeling duration 1500 ms, post-labeling delay 2025 ms).

### BOLD signals processing

Data were pre-processed using SPM8^1^ and the Data Processing Assistant for Resting-State fMRI toolbox33 (DPARSF)^2^ (Chao-Gan and Yu-Feng, 2010). The first 10 volumes of functional images were discarded, and the remaining images were then corrected for temporal shifts caused by head motion (using 24 motion-related regressions) and slice acquisition (using a least-squares approach). The realigned images were then spatially normalized to the MNI template. The resulting normalized functional images were z-transformed and smoothed with a Gaussian kernel (FWHM = 8 mm). Finally, a band-pass filter was applied to retain frequencies between 0.01 and 0.1 Hz to preserve low-frequency fluctuations. The ALFF was calculated for the filtered images using the RESTplus toolkit (V1.24)^3^ (Jia et al., 2019). The main steps involved transforming the pre-processed time series into the frequency domain using Fast Fourier Transform to compute the square root of the power spectrum for each frequency. A temporal band-pass filter (0.01–0.1 Hz) was then applied to remove low-frequency drift and physiological high-frequency noise. Finally, the individual ALFF maps were normalized using the Fisher Z-transformation. Based on previous literature and our group’s experience, ALFF shows better retest reliability and stability compared to fALFF; therefore, only ALFF calculations were performed, excluding fALFF.

### PCASL images processing

Individual raw CBF images were generated automatically using the GE workstation. The raw images underwent a visual screening process to exclude those with incomplete whole-brain coverage, significant head motion artifacts, or other conditions that did not meet the inclusion criteria. The excluded raw CBF images were processed using SPM12, ASLtbx,^4^ and MATLAB with the following key steps: (1) format conversion to NII; (2) alignment to the corresponding high-resolution T1-3D image of each individual to correct for partial volume effects; (3) spatial standardization to the MNI standard space; (4) whole-brain normalization using Fisher Z-transformation to correct for variance of individual CBF maps; and (5) spatial smoothing using a 6-mm FWHM smoothing kernel suitable for subsequent statistical analysis.

### NVC analysis

The NVC was calculated using the Multimodal Image Coupling Analysis (MICA) toolkit.^5^ Globally, the NVC was determined by calculating the correlations between vicarious neuronal activity images (ALFF maps) and regional CBF maps across the entire gray matter mask for each subject, as outlined in Hu et al. (2021). Regionally, NVC was computed by calculating the correlations between ALFF maps and regional CBF maps within each voxel of the automatic anatomical labeling 2 (AAL2) atlas region (Rolls et al., 2015), The AAL2 atlas divides the human brain into 116 regions, each with a unique label and identifier number. This analysis resulted in 116 ALFF-CBF coefficients representing the NVC within the given sub-regions.

### White matter lesions mapping

White matter lesion burden can be assessed using a conventional T2-weighted fluid-attenuated inversion recovery (T2-FLAIR) sequence. The white matter of the brain is typically divided into two regions: (1) periventricular and (2) deep subcortical white matter. Baseline WML refers to a hyperintense lesion on T2-FLAIR within the white matter. Various white matter abnormalities were assessed, including (1) total WML volume, which is the sum of individual lesion volumes, (2) WML in the right and left hemispheres, and (3) subcortical WML. WML lesions were delineated on 3D anatomical T1 and T2 FLAIR images using ITK-Snap software (version 3.8).^6^ ITK-Snap’s regional competition preprocessing function was then applied using 5 tissue clusters, 0.5 regional competition, and 0.5 smoothing force. The initial lesion segmentation was manually refined based on lesion characteristics and 3D visualization. Consensus was reached among three experts blinded to the radiologic diagnosis. In cases of disagreement, a senior neuroradiologist with more than 10 years of experience in neuroimaging was consulted for final consensus. In addition, the Fazekas score was used in this study for graded assessment of WML (Fazekas et al., 1987).

### Association analysis

Neurobehavioral associations between NVC, WML burden, and global cognition (MoCA scores) were evaluated through *Pearson’s* linear correlations, with a significance level of *p* < 0.05, *Bonferroni’s* corrected.

### Mediation analysis

Mediation analysis is a statistical approach used to examine the indirect effects of an independent variable on a dependent variable through a third variable, known as the mediator variable. In this study, the independent variable is WML burden, the dependent variable is global cognition, and the mediator variable is NVC in specific brain regions. We conducted mediation analyses using model 4 in Hayes’ SPSS macro-PROCESS (ver3.4), controlling for covariates of no interest such as TIV, age, gender, and education.

In this model, NVC within the significant regions (identified by AAL labels) served as a mediator (M), and WML burden (X) influenced the outcome of global cognition (Y). There were three pathways: (1) a direct pathway represented by c’, and two indirect pathways denoted by (2) a and (3) b. We used a bootstrapping method with 10,000 samples and a 95% confidence interval (CI) to assess the significance of the indirect effects. A 95% CI that does not include zero indicates a significant indirect effect.

### Statistical analyses

Demographic and clinical data and global NVC were analyzed using SPSS software (SPSS 22.0, Inc., Chicago, IL, USA). One-way analysis of variance (ANOVA) and Mann-Whitney *U*-tests were used to compare differences between SVCI, PSCI, and HCs for continuous variables, adjusting for data distribution and homogeneity of variance. Chi-squared tests were used for categorical variables. The threshold for statistical significance was set at *p* < 0.05.

For ALFF, CBF, and GM volume images, voxel-wise statistical analyses were performed using SPM12. These analyses were restricted to the gray matter mask, controlling for age and sex as mixed regression variables. Cluster-level family wise error (FWE) correction was applied to these imaging measures (voxel *p* < 0.001 and cluster *p* < 0.05). Surviving brain regions were visualized on cortical surfaces using BrainNetViewer software^7^.

Two-tailed, two-sample Student’s *t*-tests were performed for regional NVC. The resulting *p*-values were corrected using a false discovery rate (FDR) strategy with a threshold of *p* < 0.05.

## Results

### Demographics and neuropsychological assessment

Demographic characteristics, neuropsychological test results, and WML burden for each group are shown in Table 1. There were no significant differences between the three groups in terms of age, gender, education, hypertension, diabetes, hyperlipidemia, smoking, or alcohol consumption (*p* > 0.05). However, both PSCI and SVCI patients exhibited lower global cognitive scores on MoCA and MMSE, as well as a higher WML burden compared to the HCs group (*p* < 0.05) (Table 1).

**TABLE 1:** Demographic and clinical characteristics of the subjects.

	SVCI (*n* = 38)	PSCI (*n* = 34)	HCs (*n* = 43)	*F/X^2^/t(p)*
Gender (male/female)	30/8	27/7	33/10	0.226 (0.798)
Age (years)	60.81 ± 8.42	60.88 ± 8.68	58.23 ± 4.38	1.741 (0.180)
Education (years)	9.83 ± 2.08	9.64 ± 1.73	10.41 ± 2.30	0.156 (0.856)
Hypertension	20 (52%)	17 (50%)	16 (37%)	2.229 (0.328)
Diabetes	15 (39%)	13 (38%)	11 (25%)	2.14 (0.343)
Hyperlipidemia	13 (34%)	11 (32%)	12 (27%)	0.397 (0.819)
Smoke	22 (57%)	20 (58%)	23 (53%)	0.263 (0.876)
Alcohol	16 (42%)	17 (50%)	19 (44%)	0.909 (0.634)
MMSE	24.18 ± 2.27	21.94 ± 2.42	27.38 ± 1.21	31.36 (<0.01**)
MoCA	23.10 ± 2.34	20.88 ± 2.39	25.07 ± 2.23	49.12 (<0.001**)
WML load, ml ± SD	5.25 ± 1.65	6.32 ± 2.21	0.45 ± 0.71	−6.76 (< 0.01**)
Periventricular WML load, ml ± SD	2.88 ± 1.32	3.47 ± 2.34	0.24 ± 0.47	−7.35 (< 0.01**)
Subcortical WML load, ml ± SD	0.84 ± 1.21	2.04 ± 2.65	–	−3.48 (0.01**)
Fazekas score [median (range)]	1 (0–3)	2 (0–3)	1 (0–2)	−4.52 (0.04*)

Data are presented as mean ± SD. SVCI, small vessel disease cognitive impairment; PSCI, post-stroke cognitive impairment, HC, healthy controls; MMSE, Mini-Mental state examination; MoCA, Montreal cognitive assessment; WML, white matter lesion; a, *p*-value was obtained using the two-tailed Chi-squared test; Lesion burden corrected (corrected by total cranial volume).

**p < 0.01; *p < 0.05.

### Between-group comparison of CBF and ALFF

Supplementary Figure 1 displays the average spatial patterns of CBF for each group. Both PSCI and SVCI patient exhibit modified regional CBF in the somatomotor and default mode network (DMN) regions. Specifically, compared to the subjects in the HCs group, those with PSCI display increased perfusion in the right ventral posterolateral thalamus and decreased perfusion in the left inferior parietal, left inferior frontal, and left middle frontal gyri. Conversely, the SVCI group shows increased perfusion in the right hippocampus and vermis, along with decreased perfusion in the right middle frontal and superior temporal gyri. Moreover, the SVCI group demonstrates higher regional CBF in the left middle cingulate and posterior gyrus (refer to Supplementary Table 1). Supplementary Figure 2 illustrates the average spatial distributions of ALFF for each group. PSCI and SVCI patient exhibit altered regional ALFF in the language network and DMN regions.

### Between-group comparisons on NVC

**FIGURE 1:**
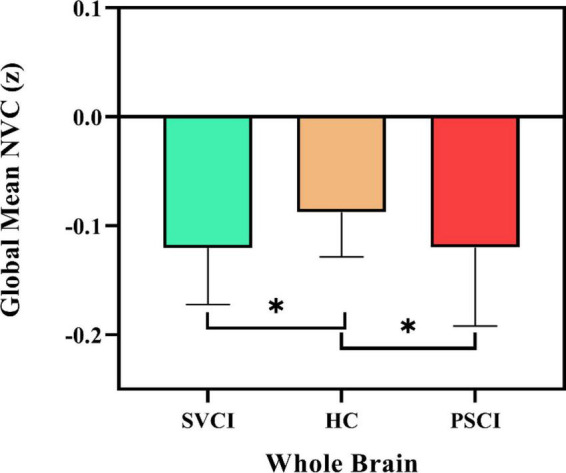
Global neurovascular coupling. Reduced global ALFF-CBF coupling in SVCI and PSCI patients, compared to HCs. At the group level, the mean of global ALFF-CBF coupling is, respectively, significantly lower (*p* = 0.009) in patients with SVCI (*p* = 0.002) and PSCI (*p* = 0.022) than in healthy controls. Error bars represent the standard error; SVCI, small vessel disease cognitive impairment; PSCI, post-stroke cognitive impairment, HC, healthy controls; NVC, neurovascular coupling. *p < 0.05.

We first delineated the global NVC patterns of the entire brain using the gray matter mask, as shown in Figure 1. Notably, both the PSCI and SVCI cohorts showed significantly reduced global NVC, with the PSCI group being among the lowest.

**FIGURE 2:**
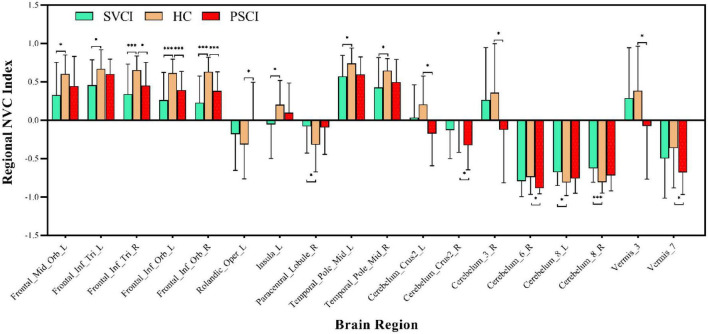
Regional neurovascular coupling. ALFF-CBF coupling differences between every two groups of SVCI, PSCI, and HCs (*p* < 0.05, FWE corrected). Compared to HCs, the SVCI patients had decreased NVC coefficient in the left middle frontal gyrus, left inferior frontal gyrus, left insula, **(middle)** temporal gyrus, and cerebellum; increased NVC coefficient in the **(right)** paracentral lobule and cerebellum while PSCI patients had decreased NVC coefficient in the right middle frontal gyrus, left inferior frontal gyrus (orbital part), **(left)** rolandic operculum, cerebellum, and vermis; SVCI, small vessel disease cognitive impairment; PSCI, post-stroke cognitive impairment, HC, healthy controls; NVC, neurovascular coupling. *p < 0.05; ^***^p < 0.001.

We then quantified cross-voxel NVC within each region defined by the AAL 116 atlas, as shown in Figure 2. Significantly divergent regions of interest (ROIs) included the left superior frontal gyrus, bilateral inferior frontal gyrus, left rolandic operculum, left insula, right paracentral lobule, middle temporal gyrus, crus II of the cerebellar hemisphere, lobule III VI VIII of the cerebellum, and lobule III VII of the vermis (*p* < 0.01, FDR corrected) (Table 2).

**TABLE 2:** Comparisons of NVC at regional level.

Brain region	PSCI	SVCI	HCs	SVCI vs. HCs *t(P)*	PSCI vs. HCs *t(P)*
Frontal_Mid_Orb_L	0.440 ± 0.389	0.326 ± 0.427	0.601 ± 0.246	−3.453 (0.019*)	—
Frontal_Inf_Tri_L	0.601 ± 0.194	0.455 ± 0.330	0.667 ± 0.249	−3.162 (0.031*)	—
Frontal_Inf_Tri_R	0.450 ± 0.302	0.338 ± 0.391	0.651 ± 0.185	−4.450 (0.001^**^)	−3.391 (0.016*)
Frontal_Inf_Orb_L	0.392 ± 0.242	0.261 ± 0.360	0.612 ± 0.182	−5.638 (0.001^**^)	−4.370 (0.001^**^)
Frontal_Inf_Orb_R	0.382 ± 0.246	0.228 ± 0.346	0.629 ± 0.189	−6.283 (0.001^**^)	−4.793 (0.001^**^)
Rolandic_Oper_L	0.005 ± 0.488	−0.181 ± 0.470	−0.318 ± 0.446	—	2.962 (0.046*)
Insula_L	0.098 ± 0.385	−0.056 ± 0.442	0.202 ± 0.315	−2.947 (0.043*)	—
Paracentral_Lobule_R	−0.095 ± 0.349	−0.081 ± 0.345	0.368 ± 0.355	2.968 (0.043*)	—
Temporal_Pole_Mid_L	0.594 ± 0.229	0.569 ± 0.274	0.739 ± 0.200	−3.102 (0.033*)	—
Temporal_Pole_Mid_R	0.494 ± 0.296	0.427 ± 0.388	0.645 ± 0.157	−3.221 (0.028*)	—
Cerebelum_Crus2_L	−0.175 ± 0.418	0.031 ± 0.428	0.204 ± 0.370	—	−4.119 (0.003^**^)
Cerebelum_Crus2_R	−0.326 ± 0.319	−0.129 ± 0.372	−0.004 ± 0.415	—	−3.764 (0.010*)
Cerebelum_3_R	−0.125 ± 0.689	0.263 ± 0.682	0.358 ± 0.637	—	−3.120 (0.036*)
Cerebelum_6_R	−0.887 ± 0.070	−0.793 ± 0.200	−0.743 ± 0.221	—	−3.842 (0.010*)
Cerebelum_8_L	−0.760 ± 0.191	−0.678 ± 0.171	−0.746 ± 0.171	3.373 (0.022*)	—
Cerebelum_8_R	−0.720 ± 0.200	−0.627 ± 0.180	−0.807 ± 0.141	4.803 (0.001^**^)	—
Vermis_3	−0.075 ± 0.692	0.286 ± 0.656	0.382 ± 0.582	—	−3.057 (0.037*)
Vermis_7	−0.680 ± 0.287	−0.499 ± 0.516	−0.364 ± 0.517	—	−3.294 (0.033*)

Data were reported as mean ± SD, and significant differences were labeled with asterisks; SVCI, small vessel disease cognitive impairment; PSCI, post-stroke cognitive impairment, HCs, healthy controls. ^**^p < 0.01; *p < 0.05.

### Association analyses

**FIGURE 3:**
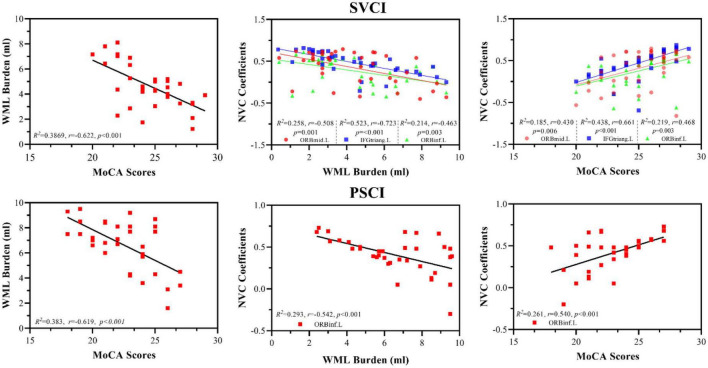
Association analyses between WML lesion, MoCA scores, and NVC; WML burden (ml) were negatively correlated with MoCA scores (SVCI patients, *R*^2^ = 0.3869, *r* = –0.622, *p* < 0.001; PSCI patients, *R*^2^ = 0.383, *r* = –0.619, *p* < 0.001) and regional NVC (SVCI patients, *R*^2^ = 0.523, *r* = –0.723, *p* < 0.001; PSCI patients, *R*^2^ = 0.523, *r* = –0.723, *p* < 0.001) in both the SVCI and PSCI groups. Furthermore, regional NVC were positively correlated with MoCA scores in both the SVCI (*R*^2^ = 0.438, *r* = 0.611, *p* < 0.001) and PSCI groups (*R*^2^ = 0.261, *r* = 0.540, *p* < 0.001) MoCA, Montréal cognitive assessment; SVCI, small vessel disease cognitive impairment; PSCI, post-stroke cognitive impairment, HC, healthy controls; NVC, neurovascular coupling; WML, white matter lesion; ORBmid.L, left middle orbital gyrus; ORBinf.L, left inferior orbital gyrus; IFGtriang.L, left triangular part of the inferior frontal gyrus.

White matter lesion burden (ml) were negatively correlated with MoCA scores (SVCI patients, *R*^2^ = 0.3869, *r* = −0.622, *p* < 0.001; PSCI patients, *R*^2^ = 0.383, *r* = −0.619, *p* < 0.001) and regional NVC (SVCI patients, *R*^2^ = 0.523, *r* = −0.723, *p* < 0.001; PSCI patients, *R*^2^ = 0.523, *r* = −0.723, *p* < 0.001) in both the SVCI and PSCI groups (Figure 3).

Furthermore, regional NVC were positively correlated with MoCA scores in both the SVCI (*R*^2^ = 0.438, *r* = 0.611, *p* < 0.001) and PSCI groups (*R*^2^ = 0.261, *r* = 0.540, *p* < 0.001) (Figure 3).

### Mediation analysis

**FIGURE 4:**
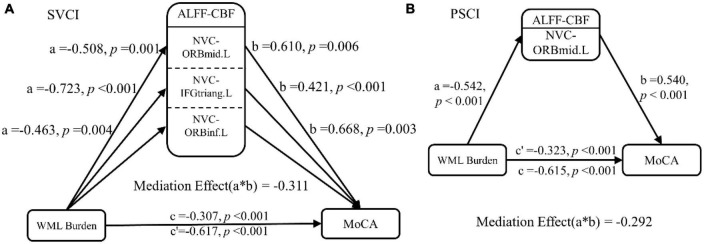
Mediation analysis. In the mediation analysis, the independent factor was the WML burden and the dependent variable was the cognitive function indicator. These were reflected by the MoCA scores, while the ALFF-CBF coefficient in the **(A)** SVCI and **(B)** PSCI patients served as the proposed mediator. The result of the mediation analysis indicated that NVC dysfunction (lower ALFF-CBF coefficient) in SVCI and PSCI patients partially mediated the effect of WML burden on global cognitive function decline. ALFF, amplitude of low frequency fluctuations; CBF, cerebral blood flow; SVCI, small vessel disease cognitive impairment; PSCI, post-stroke cognitive impairment, HC, healthy controls; NVC, neurovascular coupling; WML, white matter lesion; ORBmid.L, left middle orbital gyrus; ORBinf.L, left inferior orbital gyrus; IFGtriang.L, left triangular part of the inferior frontal gyrus.

Mediation analyses revealed that regional NVC in the left middle frontal gyrus and left inferior frontal gyrus served as partial mediators in the association between WML burden and global cognition in SVCI (c’ = −0.307, *p* < 0.001) and PSCI patients (c’ = −0.323, *p* < 0.001) (Figure 4).

## Discussion

### Main findings

This study aimed to investigate the utility of NVC as a reliable measure of cognitive decline in SVCI and PSCI patients, and its association with cognitive function and WML burden. The main findings confirmed our initial hypotheses and revealed significant correlations between NVC, cognitive test scores, and WML burden. These results support the effectiveness of NVC in examining cognitive decline in SVCI and PSCI patients. Furthermore, the study identified specific brain regions where NVC acted as a partial mediator in the relationship between WML burden and global cognition, which may indicate potential targets for interventions to improve cognitive function in these patient populations. By using multiple neuroimaging techniques, such as perfusion and functional imaging, this study provided a comprehensive understanding of the neural mechanisms underlying cognitive impairment in SVCI and PSCI patients.

### Potential physiological correlates

Neurons, including astrocytes and blood vessels, form the intricate neurovascular unit in the human brain. The integrity of this unit is critical for proper NVC (Kozberg and Hillman, 2016; Phillips et al., 2016). The connections between its components are highly complex, and damage to any element can disrupt brain structure and function, resulting in impaired NVC.

Amplitude of low frequency fluctuations represents the total power of low frequency fluctuations (0.01∼0.1 Hz), while fALFF represents the relative contribution of specific low frequency fluctuations to the total frequency range. Both ALFF and fALFF derived from BOLD signals effectively capture neuronal oxygen uptake capacity. Therefore, ALFF-CBF and fALFF-CBF analyses reveal the coordination between oxygen demand and blood supply, reflecting the functionality of the neurovascular unit (Kisler et al., 2017). In this study, 3D ASL technology provided information on CBF while rs-fMRI provided information on cerebral neural function (ALFF). The combined analysis of these two measures allowed us to investigate the impact of the disease on the neurovascular unit. This technique has been increasingly used in neuropsychiatric research (Roquet et al., 2016; Leeuwis et al., 2017). Our results indicated impaired neurovascular autonomic regulation in SVCI and PSCI patients. Notably, previous studies by Zhu et al. (2017) and Sheng et al. (2018) demonstrated reduced coupling strength in patients with schizophrenia and major depressive disorder compared with healthy subjects. Inspired by their research, the present study examined the correlation of ALFF with CBF, providing a unique perspective on this phenomenon. In addition, our study is the first to simultaneously analyze NVC in patients with both SVCI and PSCI subtypes. The observed decline in NVC in patients with PSCI and SVCI may have some impact on the treatment of cognitive impairment, as previous studies have demonstrated the efficacy of various drugs in improving NVC in animal experiments (Toth et al., 2014).

From a mechanistic perspective, the increased and decreased regions of ALFF-CBF between groups reflect different characteristics of NVC function in VCI. The comprehensive literature review highlights that NVC plays a critical role in maintaining cognitive function and that its dysfunction is associated with cognitive impairment in VCI. Specifically, the increased regions of ALFF-CBF indicate enhanced NVC, suggesting a robust coupling between neural activity and blood flow regulation. This enhancement may result from upregulated neurotransmitter release, increased vascular reactivity and improved endothelial function, facilitating efficient oxygen and nutrient delivery to active brain regions. This suggests increased neural metabolic demand and effective NVC mechanisms supporting enhanced cognitive processes. On the other hand, the decreased regions of ALFF-CBF indicate impaired NVC, where the coordination between neural activity and blood flow is compromised. This dysfunction may result from reduced neurotransmitter release, impaired vascular reactivity or endothelial dysfunction, leading to inadequate blood supply to the affected brain regions. As a result, neuronal activity may be dampened and metabolic support may be inadequate, resulting in reduced spontaneous neuronal activity and impaired cognitive function. The altered ALFF-CBF patterns in these regions provide valuable insights into the complex relationship between neural activity and vascular responses. Understanding the underlying mechanisms driving these changes is critical to unraveling the neurophysiological basis of cognitive impairment. Further investigations could explore factors such as vascular remodeling, alterations in neurovascular signaling pathways or disturbances in the neurovascular unit that may contribute to the observed changes in NVC.

In line with previous research, both cohorts of VCI patients in our study exhibited a notable increase in WML burden, particularly around the ventricles. Moreover, PSCI patients displayed additional cortical and subcortical damage. The distribution of these injuries aligned with findings from previous studies involving similar subject populations (Meyer et al., 2016; Altermatt et al., 2019). However, our primary focus was to examine changes in the intrinsic activity of the brain at rest, specifically the ALFF and local CBF. These alterations primarily affected the default mode, somatosensory/motor, dorsal attention, and language networks, which diverged to some extent from previous research findings (Cai et al., 2018; Nasios et al., 2019).

### Relationship between the NVC, WML burden, and cognitive impairment

Consistent with our hypothesis, both SVCI and PSCI patients exhibit a similar NVC pattern across the entire brain, but with an overall lower level of coupling compared to HCs. This reduction in coupling supports the findings of Liu et al. (2021) and animal studies (Tarantini et al., 2017), suggesting a disrupted interaction between spontaneous neural activity and cerebral blood flow at the global level.

In PSCI and SVCI patients, mediation analysis results show that NVC in the left middle frontal gyrus and left inferior frontal gyrus partially mediate the relationship between WML load and global cognition. This suggests that NVC in these regions may contribute significantly to the cognitive deficits observed in PSCI and SVCI patients. These findings are consistent with previous research highlighting the vulnerability of the frontal lobes to the effects of WML burden and its association with cognitive impairment in individuals with cerebrovascular disease (McAleese et al., 2021).

The left middle frontal gyrus and left inferior frontal gyrus are components of the prefrontal cortex and are located within the famous Broca’s area, which is known to be involved in several cognitive processes, including attention, working memory, and executive function. Broca’s area, a classic motor language region, plays a critical role in integrating the broader neural network and is located at the top of the cortical hierarchy. The prefrontal cortex also shows strong correlations with other brain regions and plays a role in regulating cognitive control and coordination across the brain (Ray and Zald, 2012). In this study, NVC in Broca’s area showed a significant association with WML lesions and global cognition. Mediation analysis revealed that NVC in Broca’s area partially mediated the relationship between WML burden and global cognitive impairment.

The results of this study suggest that WML burden may influence prefrontal regulation of cognitive function in patients with PSCI and SVCI, and that this effect is mediated by NVC. NVC, which reflects the interplay between resting cerebral blood flow and spontaneous neural activity, provides a more direct assessment of brain dysfunction and has greater specificity than WML burden alone (Huang et al., 2021). Identifying specific brain regions, such as the left middle frontal gyrus and the left inferior frontal gyrus, that mediate the relationship between WML burden and cognitive function is critical for developing targeted interventions to improve cognitive function in PSCI and SVCI patients. Based on the results, interventions targeting the left middle frontal gyrus and the left inferior frontal gyrus hold promise for improving cognitive function in these patient populations. Potential interventions may include cognitive training programs, brain stimulation techniques such as transcranial magnetic stimulation, or pharmacological approaches aimed at enhancing neural function and plasticity in these regions.

In conclusion, employing mediation analysis to explore the involvement of NVC in mediating the correlation between WML burden and global cognition introduces a novel methodology for comprehending the neural mechanisms associated with cognitive impairment in patients diagnosed with PSCI and SVCI. Understanding the disrupted NVC mechanisms in relation to WML burden may guide the development of interventions that specifically target these neural circuits. For example, interventions focused on enhancing NVC and improving blood flow regulation in affected brain regions may help mitigate cognitive impairment in VCI patients. The identification of specific brain regions that facilitate this correlation provides valuable insight into potential targets for interventions aimed at improving cognitive function in these patient cohorts. Further investigation is warranted to validate these findings and to develop and evaluate interventions tailored to these specific brain regions.

### Brain regions and future considerations

The default mode network (DMN), which includes the posterior cingulate cortex/precuneus, medial and dorsal prefrontal cortex, temporoparietal junction, temporal lobe, and posterior inferior parietal lobe, is the most extensively studied resting-state network system (Raichle, 2015). In this study, numerous brain regions with altered CBF and ALFF were found to be located within the DMN, suggesting that it may be the primary network affected in VCI patients. In particular, the NVC coefficient showed a significant reduction in the left precentral gyrus of VCI patients. It is noteworthy that the precentral gyrus serves as a central brain region in the sensorimotor network and is actively involved in cognitive control and motor functions (Park and Friston, 2013). This finding is consistent with previous research highlighting the vulnerability of the precentral gyrus to cerebrovascular disease, which can lead to cognitive impairment (Vipin et al., 2018; Caruso et al., 2019). In addition, reduced NVC was observed in the insula, inferior parietal lobule, and middle temporal gyrus, areas associated with specific cognitive domains. For instance, the insula has been implicated in language processing (Gogolla, 2017), and hypoperfusion in this region has been linked to the severity of cognitive decline (Sun et al., 2016).

Parietal and medial temporal regions have been implicated in visuospatial attention and working memory processes (Wang et al., 2015; Lim et al., 2020), The observed decline in cognitive function in VCI patients in this study suggests that abnormal reductions in NVC within these brain regions may contribute to deficits in specific cognitive domains. Furthermore, recent studies in both humans and rodents have revealed that cerebellar regions, in addition to their role in balance and eye movements, are associated with various aspects of cognitive function, including cognitive flexibility, spatial navigation, working memory, and certain types of discriminative learning during aging and disease states (Shipman and Green, 2019; Liang and Carlson, 2020). Specifically, reduced anatomical connectivity, ALFF, and CBF have been observed in cerebellar regions of VCI patients (Yoon et al., 2013; Diciotti et al., 2017; Li et al., 2020). Consistently, the present study also observed reduced NVC in the cerebellum, further emphasizing its importance in cognitive processes.

Moreover, when analyzing brain regions, VCI patients and HCs exhibited no significant group differences in CBF and ALFF for several brain regions. However, significant differences in NVC correlation coefficients were observed. In this context, NVC correlation coefficients serve as valuable tools for identifying abnormal regions in VCI that may not be detected by single-modality analyses of CBF or ALFF using the same statistical threshold. Conversely, the NVC correlation coefficient did not show differences between the two groups in certain brain regions with significant group differences in CBF, ALFF, or both. Taken together, NVC correlation coefficients may provide complementary information for detecting pathological changes in VCI when used in conjunction with CBF and ALFF analyses. Patients with VCI exhibit reduced NVC levels in the dorsolateral prefrontal cortex and insula, regions primarily involved in cognitive control and emotion regulation processes (Richter-Levin, 2004; Opitz, 2014; Fama and Sullivan, 2015). Within these brain regions, the dorsolateral prefrontal cortex and insula have normal CBF but reduced ALFF, suggesting that the reduced CBF-ALFF correlation in these regions is mainly due to reduced ALFF. This abnormality may partially explain the cognitive and behavioral deficits associated with these regions in VCI patients. Structural imaging studies have consistently shown reduced hippocampal volume in VCI patients, which correlates with cognitive performance (Bartsch and Wulff, 2015; Li et al., 2016). Moreover, a recent functional imaging study revealed a negative correlation between resting-state hippocampal activity and cognition in VCI patients (Zhuang et al., 2020). Based on these findings, it can be hypothesized that neurovascular decoupling impairs hippocampal function, leading to cognitive decline.

In summary, the current study identified decreased NVC coefficients within higher-order brain networks involved in cognitive control and emotion regulation, while NVC coefficients within lower-order brain networks involved in sensory processing and motor regulation remained largely unchanged. These observations provide additional support for the hypothesis that neurovascular decoupling within the brain may serve as a plausible pathological mechanism underlying VCI.

### Limitations

Firstly, while a comprehensive set of imaging and neuropsychological assessments were employed to enhance specificity, it is worth mentioning that pathological confirmation was not obtained for any of the subjects, thereby making it impossible to completely exclude concurrent neurodegenerative conditions, including AD. It is important to acknowledge that the findings may not apply universally to all individuals with VCI and that further studies incorporating pathological confirmation are needed to validate and extend the current findings. Future research should include a wider range of VCI subgroups, taking into account different etiologies and lesion locations, to provide a more comprehensive understanding of NVC alterations in different VCI populations. Secondly, this study solely focused on evaluating global cognitive function and did not encompass an assessment of other specific cognitive domains, which may limit the ability to establish associations with a broader range of brain regions displaying intergroup disparities. Lastly, it is important to acknowledge that this study only represents a cross-sectional analysis, and future longitudinal investigations are warranted to gain a more comprehensive understanding of the dynamic changes in NVC, WML burden, and cognitive impairment over time.

## Conclusion

To summarize, NVC plays an important role in mediating the association between WML burden and cognitive function in VCI patients, thus serving as a crucial neural foundation for VCI. This study provides objective neuroimaging evidence that expands our understanding of the neural mechanisms underlying VCI, with a particular focus on NVC. In essence, this research highlights the importance of using innovative approaches, such as NVC, to study cognitive decline in VCI. By providing a more accurate and reliable assessment of cognitive impairment and identifying the specific neural circuits affected by WML burden, NVC has the potential to guide the development of targeted interventions aimed at improving cognitive function in this patient population.

## Data availability statement

The original contributions presented in the study are included in the article/Supplementary material, further inquiries can be directed to the corresponding authors.

## Ethics statement

The studies involving human participants were reviewed and approved by the Ethical Medicine Society of Zhongnan Hospital of Wuhan University. The patients/participants provided their written informed consent to participate in this study. Written informed consent was obtained from the individual(s) for the publication of any potentially identifiable images or data included in this article.

## Author contributions

ZR and DS collected data, designed the experiment, analyzed data, and drafted the manuscript. LG analyzed data and revised the manuscript. HX revised the manuscript and interpreted the data. XZ and YL collected data. SL, WS, and MY provided intellectual content of critical importance to the work described. All authors also approved the version to be published.

## Footnotes

1
http://www.fil.ion.ucl.ac.uk/spm

2
http://www.restfmri.net/forum/DPARSF

3
http://www.restfmri.net/forum/RESTplus

4
https://www.cfn.upenn.edu/zewang/ASLtbx.php

5
https://github.com/hubolll/MICA_v1

6
http://www.itksnap.org

7
http://www.nitrc.org/projects/bnv
