# Supplementary material for: Altered neurovascular coupling in patients with vascular cognitive impairment: a combined ASL-fMRI analysis
Source: Front Aging Neurosci. 2023 Jun 21;15:1224525. doi: 10.3389/fnagi.2023.1224525 (PMC10320594; doi:10.3389/fnagi.2023.1224525)
Supplement: Supplementary file 1 [file Data_Sheet_1.docx]

**Supplementary Materials**

**Gray matter volume calculation**

Voxel-based morphometry (VBM) was achieved using a traditional method with Statistical Parametric Mapping (SPM12, http://www.fil.ion.ucl.ac.uk), Computational Anatomy Toolbox (CAT12.6, http://dbm.neuro.uni-jena.de/cat), and MATLAB 2013a (https://www.mathworks.com). First, the anatomical images were visually examined and then pre-processed through non-uniformity correction, skull peeling, and registration using lie algebra template to different morphological, anatomical registration. The registered images were segmented into GM and white matter (WM) classes. The GM images were then spatially normalized into Montreal Neurological Institute (MNI) space (voxel-size 1.5*1.5*1.5 mm^3^). The normalized images were modulated with a Jacobian determinant and smoothed with an 8 mm full-width-at-half-maximum (FWMH) Gaussian kernel.

**Between-group comparisons of ALFF**

Specifically, compared to the HC subjects, the PSCI exhibited increased ALFF in the superior occipital gyrus, right inferior occipital gyrus, right Anterior cingulate cortex, left fusiform gyrus, left superior frontal gyrus and left superior frontal gyrus and decreased perfusion in the cerebellum, vermis, right medial orbital gyrus, right insula, right middle frontal gyrus, left inferior frontal gyrus, left IFG pars orbitalis, right supramarginal gyrus, left middle occipital gyrus, and left middle temporal gyrus; in contrast, the SVCI group showed increased ALFF in the right middle occipital gyrus, right inferior occipital gyrus, left superior occipital gyrus and right anterior cingulate cortex and decreased ALFF in the left inferior frontal gyrus (orbital part), insula, medial orbital gyrus, right anterior cingulate cortex, cerebellar, right supramarginal gyrus, and right angular gyrus.

***
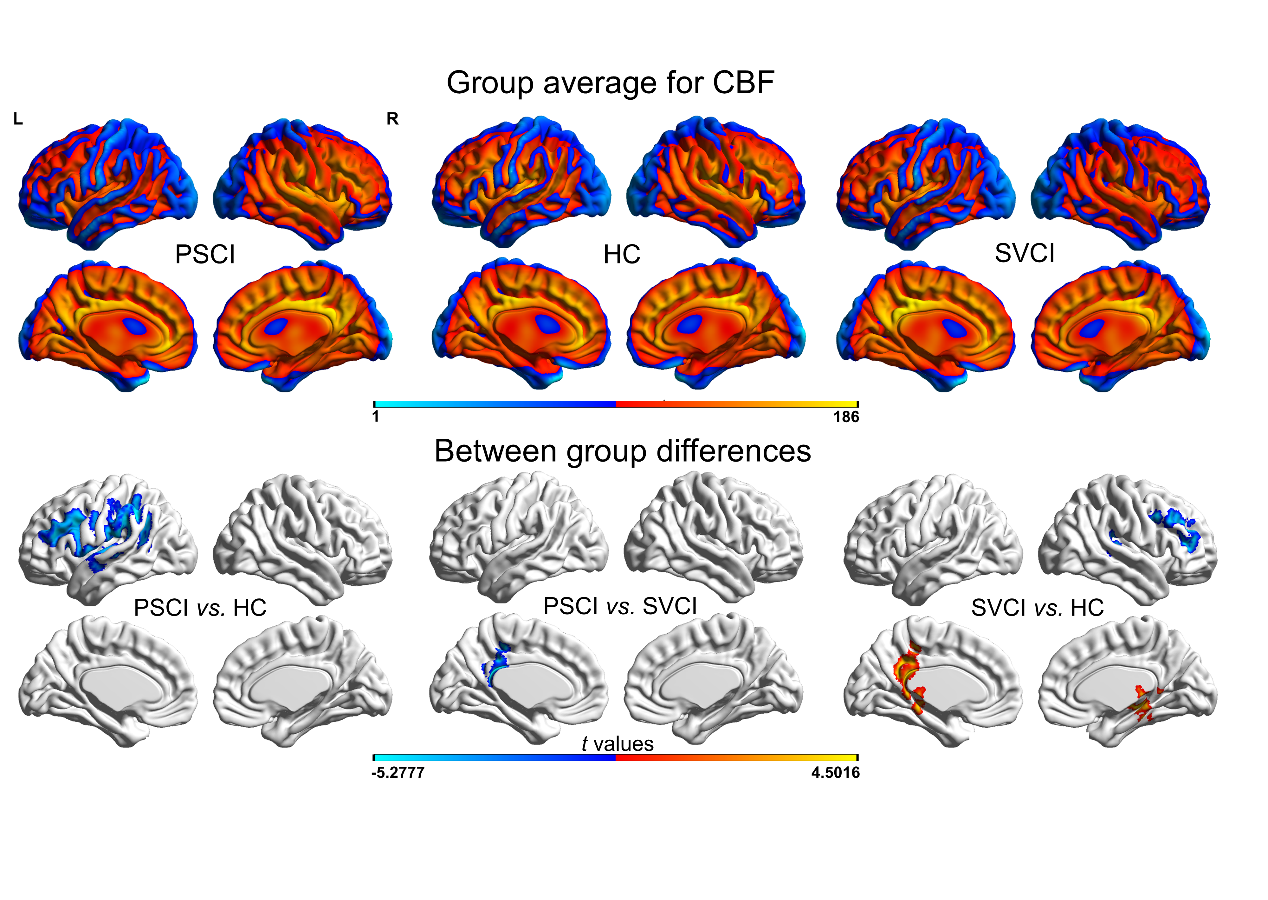
***

A

B

**Figure S1.** (A) Spatial distribution of averaged CBF in PSCI, HC, and SVCI patients. These maps were averaged across subjects within each group. (B) Group differences in CBF between patients with PSCI, SVCI, and HC (*p*<0.05, cluster FWE corrected).

**Table S1.** Brain areas with significantly different CBF among PSCI patients, SVCI patients and HC (*p* <0.05, FWE voxel-wise corrected).

| **Contrast Items** | **Region Label** | **Extent** | **t-value** |  | **MNI Coordinates** |  |
| --- | --- | --- | --- | --- | --- | --- |
|  |  |  |  | **x** | **y** | **z** |
| Positive |  |  |  |  |  |  |
| PSCI>HC | Thal_VPL_R | 161 | 3.798 | 22 | -24 | 2 |
|  |  |  |  |  |  |  |
| SVCI>HC | Hippocampus_R | 2768 | 4.5016 | 24 | -30 | -8 |
|  | Vermis_3 | 2768 | 4.2693 | 2 | -46 | -20 |
| Negative |  |  |  |  |  |  |
| PSCI<SVCI | Cingulate_Post_L | 402 | -4.1954 | -8 | -42 | 18 |
|  | Cingulate_Mid_L | 402 | -3.8178 | -8 | -34 | 44 |
|  |  |  |  |  |  |  |
| SVCI<HC | Frontal_Mid_2_R | 821 | -4.8306 | 36 | 44 | 28 |
|  | Frontal_Mid_2_R | 821 | -4.0784 | 42 | 24 | 36 |
|  | Temporal_Sup_R | 233 | -4.2599 | 44 | -32 | 14 |
|  | Frontal_Mid_2_R | 178 | -4.1276 | 44 | 10 | 38 |
|  |  |  |  |  |  |  |
| PSCI<HC | Parietal_Inf_L | 5827 | -5.2777 | -54 | -40 | 40 |
|  | Frontal_Inf_Tri_L | 5827 | -4.7051 | -46 | 32 | 24 |
|  | Frontal_Mid_2_L | 5827 | -4.6619 | -42 | 12 | 34 |

Table S1 shows all local maxima. x, y, and z = MNI coordinates. Abbreviations: T-value: the voxel of maximal intensity in this cluster; MNI: Montreal Neurological Institute**;** x, y, z: coordinates of primary peak locations in the MNI space. SVCI, small vessel disease patients; PSCI, large vessel disease patients; HC, healthy controls. The threshold was set at voxel p< 0.05, FWE corrected.


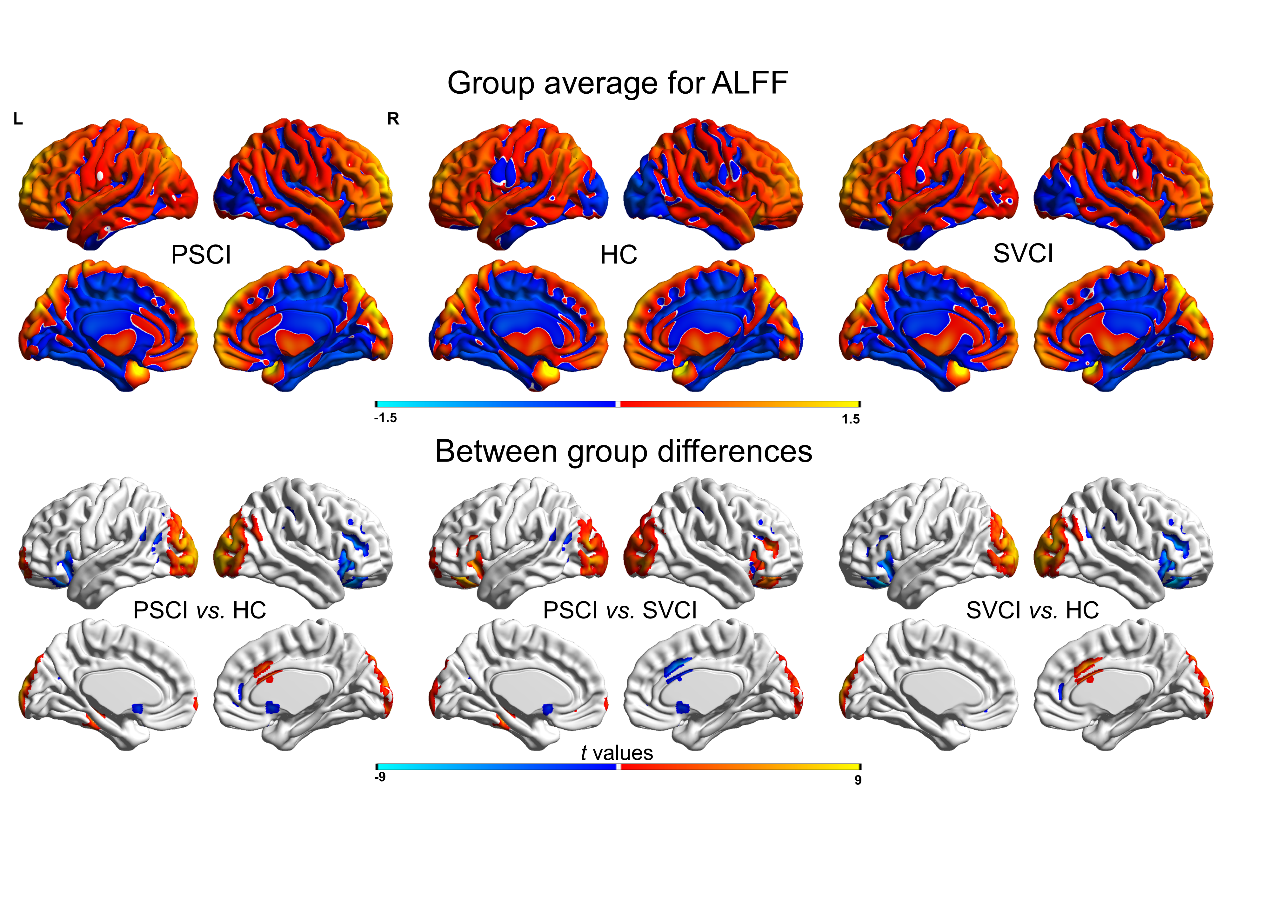


**Figure S2.** (A) Spatial distribution of averaged ALFF in PSCI, SVCI patients and HC. These maps were averaged across subjects within each group. (B) Group differences in ALFF between patients with PSCI, SVCI, and HCs (*p*<0.05, cluster FWE corrected).

**Table S2.** Brain areas with significantly different ALFF between PSCI patients vs. SVCI patients vs. HC (*p* < 0.05, FWE corrected).

| **Contrast items** | **Region Label** | **Extent** | **t-value** | **MNI Coordinates** | | |
| --- | --- | --- | --- | --- | --- | --- |
|  |  |  |  | **x** | **y** | **z** |
| Positive |  |  |  |  |  |  |
| PSCI>HC | Occipital_Sup_L | 1607 | 9.144 | -12 | -102 | 18 |
|  | Occipital_Sup_R | 1607 | 8.403 | 21 | -102 | 3 |
|  | Occipital_Inf_R | 1607 | 8.398 | 36 | -96 | -9 |
|  | ACC_sup_R | 86 | 7.089 | 9 | 15 | 24 |
|  | Fusiform_L | 100 | 5.692 | -39 | -33 | -21 |
|  | Frontal_Sup_2_L | 86 | 5.574 | -24 | 66 | -9 |
|  | Frontal_Sup_Medial_L | 86 | 4.264 | -3 | 72 | 3 |
|  |  |  |  |  |  |  |
| PSCI>SVCI | Occipital_Mid_L | 1367 | 3.131 | -42 | -87 | 9 |
|  | Cuneus_L | 1367 | 2.660 | 3 | -99 | 15 |
|  | Occipital_Inf_R | 1367 | 2.433 | 42 | -90 | -6 |
|  | OFCpost_L | 645 | 2.891 | -27 | 30 | -15 |
|  | Insula_L | 645 | 2.118 | -30 | 27 | 6 |
|  | Frontal_Sup_2_L | 645 | 1.481 | -27 | 24 | 33 |
|  | OFCpost_R | 776 | 2.226 | 36 | 33 | -21 |
|  | Frontal_Mid_2_R | 776 | 1.984 | 45 | 39 | 15 |
|  | ParaHippocampal_L | 99 | 2.207 | -30 | -33 | -15 |
|  | Frontal_Sup_Medial_L | 83 | 1.587 | -3 | 69 | 0 |
|  | Frontal_Sup_2_L | 83 | 1.002 | -24 | 63 | -6 |
|  |  |  |  |  |  |  |
| SVCI>HC | Occipital_Sup_L | 1512 | 8.569 | -15 | -102 | 15 |
|  | Occipital_Inf_R | 1512 | 7.921 | 27 | -102 | -3 |
|  | Occipital_Mid_R | 1512 | 7.547 | 42 | -87 | 15 |
|  | ACC_sup_R | 123 | 7.371 | 12 | 18 | 27 |
| Negative |  |  |  |  |  |  |
| PSCI<HC | Cerebellum_8_R | 1519 | -9.376859665 | 24 | -66 | -60 |
|  | Cerebellum_8_L | 1519 | -8.98973465 | -27 | -66 | -57 |
|  | Vermis_8 | 1519 | -4.441483498 | 3 | -60 | -27 |
|  | OFCmed_R | 736 | -9.109071732 | 18 | 24 | -18 |
|  | Insula_R | 736 | -8.008119583 | 30 | 27 | 6 |
|  | Frontal_Mid_2_R | 736 | -6.103502274 | 24 | 30 | 27 |
|  | Frontal_Inf_Orb_2_L | 365 | -7.832396507 | -21 | 27 | -12 |
|  | Frontal_Inf_Tri_L | 365 | -3.961125135 | -36 | 36 | 0 |
|  | SupraMarginal_R | 99 | -5.417437553 | 42 | -36 | 39 |
|  | Occipital_Mid_L | 222 | -4.722148418 | -30 | -69 | 27 |
|  | Temporal_Mid_L | 222 | -4.378072262 | -39 | -54 | 12 |
|  |  |  |  |  |  |  |
| PSCI<SVCI | Cerebellum_8_L | 1532 | -3.226455212 | -12 | -69 | -39 |
|  | Cerebellum_8_R | 1532 | -3.116758823 | 12 | -69 | -36 |
|  | Cerebellum_7b_R | 1532 | -1.542641163 | 48 | -60 | -57 |
|  | Cingulate_Mid_R | 118 | -2.882619619 | 9 | 3 | 36 |
|  | Occipital_Mid_L | 238 | -2.837670088 | -33 | -69 | 24 |
|  | Temporal_Mid_L | 238 | -1.043218374 | -39 | -51 | 9 |
|  | N_Acc_R | 776 | -2.247002602 | 12 | 18 | -9 |
|  | Frontal_Sup_2_R | 776 | -1.579194665 | 24 | 30 | 30 |
|  | Frontal_Inf_Tri_R | 776 | -0.873274326 | 42 | 21 | 12 |
|  | Postcentral_R | 87 | -2.027133703 | 33 | -30 | 39 |
|  |  |  |  |  |  |  |
| SVCI<HC | Frontal_Inf_Orb_2_L | 651 | -9.479067802 | -21 | 27 | -12 |
|  | Insula_L | 651 | -6.94493866 | -27 | 27 | 12 |
|  | OFCmed_R | 743 | -9.22209549 | 18 | 24 | -18 |
|  | Insula_R | 743 | -8.798873901 | 33 | 27 | 3 |
|  | ACC_pre_R | 743 | -5.758385658 | 15 | 39 | 0 |
|  | Cerebellum_8_R | 364 | -8.048294067 | 24 | -69 | -60 |
|  | Cerebellum_8_L | 382 | -7.510435581 | -24 | -69 | -57 |
|  | SupraMarginal_R | 86 | -4.335533619 | 42 | -33 | 39 |
|  | Angular_R | 86 | -4.068728924 | 45 | -51 | 27 |

Table S2 shows all local maxima. x, y, and z = MNI coordinates. Abbreviations: BA: Brodmann’s area; t-value: the voxel of maximal intensity in this cluster**;** x, y, z: coordinates of primary peak locations in the MNI space. The threshold was set at voxel p <0.05, FWE corrected. ALFF, amplitude of low-frequency fluctuation.
